# Confabulating, Misremembering, Relearning: The Simulation Theory of Memory and Unsuccessful Remembering

**DOI:** 10.3389/fpsyg.2016.01857

**Published:** 2016-11-25

**Authors:** Kourken Michaelian

**Affiliations:** Department of Philosophy, University of OtagoDunedin, New Zealand

**Keywords:** episodic memory, confabulation, DRM effect, causal theory of memory, simulation theory of memory

## Abstract

This article develops a taxonomy of memory errors in terms of three conditions: the accuracy of the memory representation, the reliability of the memory process, and the internality (with respect to the remembering subject) of that process. Unlike previous taxonomies, which appeal to retention of information rather than reliability or internality, this taxonomy can accommodate not only misremembering (e.g., the DRM effect), falsidical confabulation, and veridical relearning but also veridical confabulation and falsidical relearning. Moreover, because it does not assume that successful remembering presupposes retention of information, the taxonomy is compatible with recent simulation theories of remembering.

Memory errors play important roles in both psychology and philosophy. In psychology, they provide insight into the mechanisms at work in successful and unsuccessful remembering (e.g., Roediger, 1996). In philosophy, they serve as test-cases for accounts of the difference between genuine and merely apparent remembering (e.g., Bernecker, 2010). But there is little consensus in either psychology or philosophy on the definitions of and relationships among different types of memory error—we lack an adequate taxonomy of memory errors.

Drawing on the resources of philosophy but aiming for coherence with psychology, this article develops a taxonomy designed to be consistent with recent simulation theories of remembering. While such theories are increasingly influential (Shanton and Goldman, 2010; De Brigard, 2014a; Michaelian, 2016b), the older causal theory of memory Martin and Deutscher (1966) remains popular (e.g., Bernecker, 2010; Klein, 2015; Cheng and Werning, 2016; Debus, Forthcoming). Indeed, basing her approach in part on the causal theory, Robins (2016a) has recently proposed a taxonomy of memory errors in terms of two conditions: (1) the accuracy of the memory representation and (2) the involvement of retained information in the production of that representation. This article begins by arguing that, while the causalist taxonomy accommodates several types of error, including misremembering, falsidical confabulation, and veridical relearning, it fails to accommodate others, namely, veridical confabulation and falsidical relearning. The article then develops an alternative simulationist taxonomy in which the reliability of the memory process that produced the representation replaces the involvement of retained information in the production of the representation. This initial simulationist taxonomy accommodates misremembering and (veridical and falsidical) confabulation, but it does not provide an account of (veridical and falsidical) relearning. The article therefore ultimately proposes a taxonomy of memory errors in terms of three factors: (1) accuracy, (2) reliability, and (3) the “internality” (with respect to the remembering subject) of the memory process. This taxonomy provides a plausible account of the relationships among remembering and misremembering, veridical and falsidical confabulation, and veridical and falsidical relearning, while remaining consistent with the simulation theory.

Before proceeding, it is worth briefly addressing two worries that one might have about the general sort of taxonomy of memory errors at issue here, whether simulationist or causalist. First, one might suppose that, since a great deal is known about the mechanisms underlying confabulation and other types of memory error, we would be better off dispensing with highly general conditions such as accuracy, reliability, and internality and instead defining memory errors directly in terms of their underlying neural bases. Given the wide variety of conditions under which even a single type of memory error may arise, however, it is unlikely to be feasible to construct such a taxonomy (Hirstein, 2005). Second, recent accounts of memory errors (e.g., Bortolotti and Cox, 2009; Fotopoulou, 2010; Droege, 2015; Fernández, 2015) have tended to emphasize their potential adaptive benefits, and one might argue that the accounts discussed here do not acknowledge such benefits. The point of these taxonomies is not, however, to determine whether or not memory errors are adaptive. The point is rather to contribute to the conceptual clarity of investigations of the adaptive significance and underlying mechanisms of various types of memory errors.

## 1. The causalist taxonomy

The starting-point for Robins' development of the causalist taxonomy is the contrast between *confabulation*^1^ and (what she refers to as) *misremembering*.

### 1.1. Confabulation and misremembering

Robins takes confabulation, on the one hand, to be exemplified by “lost in the mall”-style experiments, in which subjects are induced to remember events that they never experienced (Loftus and Pickrell, 1995). This sort of memory error, she says, reflects “no influence of retained information from a particular past event” (Robins, 2016a, p. 434). Since confabulated memory representations may be built up from components originating in experience of different past events, what is intended here is clearly that confabulation reflects no influence of retained information from *the* particular past event described by the confabulated representation. She takes misremembering, on the other hand, to be exemplified by the Deese-Roediger-McDermott (DRM) effect, in which subjects who have studied a list of thematically-related words remember non-presented but thematically-consistent words (Deese, 1959; Roediger and McDermott, 1995). This sort of memory error, according to Robins, “relies on successful retention of the targeted event” (Robins, 2016a, p. 434).

This way of describing the contrast between confabulation and misremembering is meant to make it analogous to the contrast between perceptual hallucination, in which the subject forms a representation which does not correspond to the scene before his eyes, and perceptual illusion, in which the subject forms a representation which does correspond to the scene before his eyes in some respects but fails to correspond to it in others. The analogy between the confabulation/misremembering distinction and the hallucination/illusion distinction will play an important role in the argument of Sections 2–3. For now, the point is that, as Robins conceives of it, misremembering involves a combination of success and failure: the subject retains information from experience of the remembered event (success) but nevertheless represents the event inaccurately (failure)^2^. Because it involves this specific combination of success and failure, she claims, neither the traditional causal theory of memory—according to which successful remembering presupposes the existence of a causal connection between the retrieved memory representation and an earlier experience of the event it represents—nor more recent constructive theories is able to explain its occurrence.

Traditional causal theorists adopt an essentially archival view of memory, seeing it as “a preservative capacity that stores discrete representations of particular past events” (Robins, 2016a, p. 432). Since the archival view treats remembering as being simply a matter of retrieving stored representations, it is able to understand memory errors only as resulting from *malfunctions* of the memory system. It is thus unable to explain the occurrence of errors in which a representation is successfully stored, indicating that the system is functioning properly, but in which the subject nevertheless fails to form an accurate representation at the time of retrieval. This analysis of the limits of a purely archival view is persuasive; Robins' analysis of the limits of the constructive view is much less so. The constructive view, as she describes it, holds that “all attempts at remembering—both successes and errors—are outputs of a single, adaptive process by which plausible representations are constructed at the time of recall” (Robins, 2016a, p. 432). Distinguishing among several versions of the constructive view, including connectionist (Sutton, 1998), gist-based (Michaelian, 2011b), and simulationist [or what she refers to as episodic hypothetical thought-based; (De Brigard, 2014a)] approaches, Robins argues that, despite the fact that it was expressly designed to take memory errors into account, the constructive view, like the archival view, is unable to explain the occurrence of the particular error of misremembering.

Simulationism is increasingly prominent, and the focus here will be on the ability of this particular version of the constructive view to account for misremembering and other errors. A number of distinct simulationist approaches have been proposed, but they have in common that they see memory as a special case of a more general capacity for mental time travel (Suddendorf and Corballis, 2007), episodic hypothetical thought (De Brigard, 2014a), or episodic imagination more broadly (Michaelian, 2016b). The core idea is that, in episodic remembering, the episodic memory system—or rather a broader episodic construction system—draws on information acquired during experience of past events to construct a simulation of a target event from the subject's personal past. Similarly, in episodic future thinking, the episodic construction system draws on information acquired during experience of past events to construct a simulation of a future event and, in episodic counterfactual thinking, the system draws on such information to construct a simulation of a counterfactual past event. Just as the system must be able to simulate a future or counterfactual event without drawing on information originating in experience of the target event (since the event has not been experienced), it is able to simulate a past event without drawing on information originating in experience of the event (though, since the event has been experienced, it may do so). Successful remembering, according to simulationism, does not presuppose the retention of information from the subject's experience of the remembered event, and therefore—contra the causal theory—it does not presuppose the existence of a causal connection between a “retrieved” memory representation and an earlier experience of the event it represents^3^.

Whereas the traditional causal theory is only able to see memory errors as resulting from malfunctions of the system, leaving it unable to explain the occurrence of misremembering, Robins takes it that simulationism is only able to see memory errors as byproducts of an imperfect but *properly functioning* system, leaving it likewise unable to explain the occurrence of misremembering. For the simulationist, neither successful nor unsuccessful remembering need involve retention of a discrete representation of the remembered event, but “[w]ithout appeal to [a discrete representation], there is no way to constrain one's consideration of which details were most likely part of that event” (Robins, 2016a, p. 443). Simulationism thus fails to acknowledge the difference between misremembering, which involves retention of information originating in experience of the remembered event, and outright confabulation, which does not.

### 1.2. The accuracy and retention conditions

Seeking a compromise between the archival view and the constructive view, Robins proposes a hybrid view. From the archival view, she takes the idea that successful remembering involves *retention of information* from the remembered event. From the constructive view, she takes the idea that successful remembering involves *construction of an accurate representation* at the time of retrieval. These two conditions—retention and accuracy—ground an intuitively appealing account of the relationship between successful remembering, misremembering, and confabulation. *Successful remembering* occurs when both the retention condition and the accuracy condition are met. *Misremembering* occurs when the retention condition is met but the accuracy condition is not. Finally, *confabulation* occurs when neither condition is met.

It is important to note that, while it may not be compatible with traditional archival versions of the causal theory, Robins' taxonomy is explicitly intended to be compatible with newer constructive versions of the theory. Indeed, setting aside their disagreement over the nature of memory traces, Robins' retention condition is equivalent to the conjunction of Martin and Deutscher's causal and memory trace conditions (see Section 2). Robins denies that successful remembering can be *reduced to* the retrieval of a stored representation; but she takes it that storage and retrieval of a representation—and hence a causal connection with the remembered event—is *essential* to successful remembering. Her taxonomy is thus causalist in character. As such, it is incompatible with the simulation theory, which denies that successful remembering requires retention of information from experience of the remembered event.

## 2. Problems for the causalist taxonomy

Were we to accept the taxonomy, we would thus have reason to abandon simulationism. But while the taxonomy may be intuitively appealing, it is ultimately inadequate. An adequate taxonomy will accommodate not only misremembering and confabulation but a number of additional memory errors, and Robins' taxonomy is unable to do so.

### 2.1. Relearning

Retention and accuracy can be combined in four ways (see Table 1). First, the subject retains information and therefore retrieves an accurate representation; this is the causalist account of successful remembering. Second, the subject retains information but nevertheless retrieves an inaccurate representation; this is the causalist account of misremembering. Third, the subject does not retain information and therefore retrieves an inaccurate representation; this is the causalist analysis of confabulation. Finally, the subject does not retain information but nevertheless retrieves an accurate representation. This final combination is not discussed by Robins in detail, but she does suggest that it corresponds to *relearning*.

**Table 1:** **The causalist taxonomy of memory errors**.

	**Retention**	**Accuracy**
Successful remembering	Yes	Yes
Misremembering	Yes	No
Relearning	No	Yes
Falsidical confabulation	No	No

Relearning, as the term is used in the relevant literature, occurs in certain cases in which the subject's memory of an event depends entirely on an external prompt. Reading an entry in one's diary, for example, might enable one to remember an event that one would otherwise be unable to remember. In some such cases, one might be successfully remembering the event. But in other cases, one might merely be parroting back the prompt, i.e., one might have relearnt the event. It is not immediately clear whether relearning should, strictly speaking, be classified as a memory error. Relearning (as philosophers use the term) is not among the memory errors standardly studied by psychologists. But relearning is clearly closely related to remembering, and, while it is not natural to view relearning as an error if the subject is aware that he is relearning, the same thing goes for the other errors discussed here—in the cases with which we are concerned, it is assumed that the subject takes himself to be remembering. Hence it is appropriate to include it in a taxonomy of memory errors. One desideratum for the taxonomy is thus to distinguish between cases of successful remembering and cases of relearning. Robins' suggestion is that relearning occurs when the the accuracy condition is met but the retention condition is not. This suggestion cannot be right, however, for there is another type of memory error in which the accuracy condition is met but the retention condition is not, namely, *veridical confabulation*.

### 2.2. Veridical confabulation

“Veridical confabulation” may sound like an oxymoron, because “confabulation” sounds *counterfactive*: intuitively, it seems that one can only confabulate something that is false. It is thus unsurprising that many standard definitions of confabulation build falsity into confabulation. In one early definition, Talland describes a confabulation as a “false verbal statement about facts” (Talland, 1961, p. 362). In another early definition, Berlyne describes a confabulation as a “falsification of memory occurring in clear consciousness in association with an organically derived dementia” (Berlyne, 1972, p. 38). More recently, Berrios describes confabulations along the same line as “inaccurate or false narratives purporting to convey information about world or self” (Berrios, 2000, p. 348). Similarly, Robins asumes that confabulations are inaccurate. But falsity should not be built into confabulation, for one and the same confabulatory process might lead to the formation of either an accurate (true) or an inaccurate (false) representation.

Recall the analogy between the confabulation/misremembering distinction and the hallucination/illusion distinction. In Section 1, the analogy was introduced by saying that the difference between hallucination and illusion is that, in hallucination, the subject forms a representation which does not correspond to the scene before his eyes, whereas, in illusion, the subject forms a representation which does correspond to the scene before his eyes in some respects but fails to correspond to it in others. Though natural, this way of describing the difference between hallucination and illusion is inadequate, since it overlooks the possibility of veridical hallucination, hallucination in which the subject's visual representation happens to correspond to the scene before his eyes (e.g., Lewis, 1980). Most cases of hallucination will result in inaccurate representations, so falsidical hallucination is the norm. But hallucination might occasionally result in an accurate representation, so the possibility of veridical hallucination must be accommodated.

Veridical confabulation in memory is analogous to veridical hallucination in perception. Most cases of confabulation will result in inaccurate memory representations; falsidical confabulation is the norm. But confabulation might occasionally result in an accurate memory representation; the possibility of veridical confabulation must be accommodated. In “lost in the mall” experiments, for example, subjects are normally asked to imagine events that are highly improbable but nevertheless possible. Such an experiment will result in a confabulatory but veridical representation should it turn out that the subject really had experienced the relevant event. In many cases of clinical confabulation, the confabulated representations are outright incoherent and therefore impossible. But there are also cases of clinical confabulation which produce representations which are coherent and therefore possible. Again, such a case will involve a confabulatory but veridical representation should it turn out that the subject had in fact experienced the relevant event^4^.

What is needed, then, is a definition of confabulation that does not assume that confabulated representations are inaccurate. One standard way of distinguishing between veridical hallucination and successful perception is to appeal to the absence, in the former case, and the presence, in the latter case, of a *causal connection* between the subject's representation and the scene before his eyes, and we might similarly hope that a condition requiring a causal connection between the subject's memory representation and his original experience will provide a means of distinguishing between veridical confabulation and successful remembering. Indeed, Martin and Deutscher themselves invoke the possibility of veridical confabulation to motivate a causal condition on remembering (see Robins, 2016b). The idea, as they put it, is that remembering an event requires that the subject's experience of it “must have been operative in producing a state or successive states in him finally operative in producing his representation” (Martin and Deutscher, 1966, p. 173). They later clarify that the relevant state is a *memory trace*, which they view as a “structural analog” of experience. Call a causal connection that goes via a memory trace in the relevant manner a “trace connection.” On Martin and Deutscher's approach, both veridical and falsidical confabulation are characterized by the lack of a trace connection between the memory representation and the earlier experience.

This approach might give us a way distinguish between successful remembering (which, if the causal theorist is right, involves a trace connection) and veridical confabulation (which does not). And it might distinguish between misremembering (which, again, if the causal theorist is right, involves a trace connection) and falsidical confabulation (which does not). But it does not distinguish between veridical confabulation and relearning, neither of which involves a trace connection. Martin and Deutscher do appeal to relearning to motivate a refinement of the causal condition: where prompting contributes to the production of the memory representation, the subject's experience of the relevant event “is operative in producing the state (or successive set of states) in him which is finally operative in producing the representation *in* the circumstances in which he is prompted (Martin and Deutscher, 1966, p. 185). But this just reiterates the point made by the generic causal condition: the experience must give rise to a memory trace which then contributes to the production of the memory representation, even if the prompt also contributes, i.e., there must be a trace connection between the memory representation and the earlier experience. The refined causal condition tells us how to distinguish relearning from successful remembering, but it does not tell us how to distinguish relearning from veridical confabulation.

In light of the discussion above of the limits of the causalist taxonomy, this should come as no surprise, for Robins' retention condition is roughly equivalent to Martin and Deutscher's trace connection condition. The difference between them is simply that, whereas Martin and Deutscher view traces as “structural analogs,” Robins understands them in terms of retained information. Neither the causal condition nor the retention condition provides a means of simultaneously accommodating both veridical confabulation and relearning.

### 2.3. Falsidical relearning

There is another type of memory error that the taxonomy is unable to accommodate, namely, *falsidical relearning*. The discussion so far has assumed (with Robins) that relearning is always veridical, but it can also be falsidical, i.e., it can result in inaccurate memory representations. “Falsidical relearning,” like “veridical confabulation,” may sound like an oxymoron, because “(re)learning” sounds *factive*: intuitively, it seems that one can only learn or relearn something that is true. But regardless of how the term is ordinarily used, relearning should not be treated as factive here, for the straightforward reason that one and the same relearning process might lead to the formation of either an accurate or an inaccurate representation.

Cases of falsidical relearning are easy enough to generate: the description of the diary case given above, for example, assumed that the diary contained accurate records of the subject's past, but this need not be so, and in a variant of the case in which the subject consults a diary containing inaccurate records, he will undergo falsidical relearning. Just as it cannot simultaneously accommodate veridical confabulation and veridical relearning, since both satisfy the accuracy condition but not the retention condition, the causalist taxonomy cannot simultaneously accommodate falsidical confabulation and falsidical relearning, since both satisfy neither the accuracy condition nor the retention condition.

## 3. Toward a simulationist taxonomy

One basic lesson of the argument of Section 2 is that the causalist taxonomy simply does not include enough conditions. Because it includes only two conditions—accuracy and retention—allowing for four combinations, it can in principle account for the relationships among successful remembering and at most three types of memory error. Given that an adequate taxonomy must account for the relationships among successful remembering and at least five types of error—misremembering, veridical and falsidical confabulation, and veridical and falsidical relearning—we can assume that it will include at least three conditions. One natural approach to producing such a taxonomy would be to supplement the accuracy and retention conditions with a third condition. Any taxonomy which includes the retention condition would, however, be incompatible with simulationism, and this section will therefore explore an alternative approach.

### 3.1. Confabulation

We saw above that, while it is tempting to define confabulation in terms of falsity, there is good reason not to do so. Örulv and Hydén, for example, describe confabulations as “as false narrative or statements about world and/or self due to some pathological mechanisms or factors, but with no intention of lying” (Örulv and Hydén, 2006, p. 648). This builds falsity into the definition, which we do not want to do. But it also builds in a second factor, production of the representation by a pathological mechanism or factor, and it thus begins to get at an important feature of confabulation. Consider again, the contrast between confabulation and relearning. Intuitively, veridical relearning occurs in cases in which the subject seems to remember, and to remember accurately, but in which he himself contributes nothing to the production of his memory representation, in the sense that he contributes no content to the retrieved representation. Veridical confabulation, in contrast, occurs in cases in which the subject seems to remember, and to remember accurately, but in which he is just “making things up,” in the sense that his memory representation is accurate with respect to the target event only by chance. The difference between falsidical relearning and falsidical confabulation can be described in parallel terms. Falsidical relearning occurs in cases in which the subject seems to remember, though to remember inaccurately, and in which he himself contributes nothing to the production of the memory representation, i.e., he contributes no content to the retrieved representation. Falsidical confabulation, in contrast, occurs in cases in which the subject seems to remember, though to remember inaccurately, and in which he is just making things up, in the sense that, were the memory representation accurate with respect to the target event, it would be so only by chance. From an intuitive standpoint, in other words, neither confabulation nor relearning is about retention. Confabulation is about lack of *reliability*. Relearning is about lack of *internality*.

#### 3.1.1. The reliability condition

Relearning will be discussed in Section 4. The remainder of Section 3 develops the idea that confabulation is about lack of reliability. Reliability is understood here in the epistemologist's sense: a reliable system is, roughly, one that produces mostly accurate representations. But reliability is not a purely statistical notion, since even a reliable system might by chance produce many inaccurate representations. It is thus a modal notion, and a reliable system is, more precisely, one that tends to produce mostly accurate representations, at least when operating under normal conditions.

If simulationism is right, remembering is always at least in part a matter of “making things up,” i.e., of generating a more or less probable representation of a target event. In a loose sense, then, we might say that all memories are to some extent confabulatory. But there are different ways of making things up, and, in a strict sense, we can distinguish between successful remembering and confabulation in terms of the probability that the generated representation is accurate. In a subject with a properly functioning memory system, generated representations have a high probability of being accurate. In a confabulating subject, generated representations have a low probability of being accurate. Confabulation, then, occurs when the subject's episodic memory system functions unreliably. When the system functions unreliably, it will usually produce an inaccurate representation. In cases where an unreliably functioning memory system produces an inaccurate representation, the subject can be said to confabulate falsidically. But even an unreliably functioning system might occasionally produce an accurate representation. In cases where an unreliably functioning memory system produces an accurate representation, the subject can be said to confabulate veridically^5^.

This approach to confabulation is broadly in line with Hirstein's definition in terms of the notion of “ill-groundedness,” which may itself be understood in terms of reliability (Hirstein, 2005). It is meant to apply to core cases of clinical confabulation. It is not, for example, satisfied by “lost in the mall” cases.^6^ But though such cases are cited by Robins (2016a) as characteristic examples of confabulation, they are importantly different from clinical confabulation cases, so the fact that the definition is not satisfied by them does not pose a problem. In a standard “lost in the mall”-style experiment, the subject is asked to imagine an episode in detail. Later, he mistakenly takes the resulting representation to have originated in experience rather than in imagination. Contrast this with a typical case of clinical confabulation. The case of Dalla Barba's patient SD is representative. SD had suffered severe head trauma. When asked what he had done the day before, he replied: “Yesterday I won a running race and I was awarded with a piece of meat which was put on my right knee” (Dalla Barba, 1993). This report was inaccurate, the result of inappropriately recombining elements from different events (SD had been a runner, and he had once injured his right knee during a race). “Lost in the mall” cases certainly involve a kind of memory error, but the mechanism responsible for the error is distinct from that at work in clinical confabulation cases. In both “lost in the mall” cases and clinical confabulation cases, the memory representation might turn out by chance to be accurate but will normally be inaccurate. A different explanation of the tendency of the representation to be inaccurate is required in each sort of case. The inaccuracy in “lost in the mall” cases is best described as resulting from a source monitoring (Johnson et al., 1993) or other metacognitive error. It is due to a failure at the level of metacognitive monitoring, not at the level of the process responsible for generating the representation. The inaccuracy in clinical confabulation cases, in contrast, is due to a failure at the level of the process responsible for generating the representation. It may be compounded by metacognitive failure, but it results in the first place from a failure of the episodic construction system to generate a representation that is likely to be accurate.

The point can be put in epistemological terms. On many accounts, when remembering is successful, episodic memory provides the subject with two kinds of knowledge. The remembered first-order content provides him with knowledge of the target event. And accompanying meta-level autonoetic phenomenology (Dokic, 2014) or self-reflexive content (Fernández, 2016) provides him with knowledge that the first-order content originates in his experience of the event. Episodic memory, in other words, provides the subject both with first-order knowledge of what happened in the past and with meta-level knowledge of how he knows that it happened. Bearing this characteristic feature of episodic memory in mind, it is easy to explain the inclination to assimilate “lost in the mall” cases to confabulation, for both such cases and clinical confabulation cases involve inaccuracy at both the first-order level and the meta-level: the subject forms a representation of an event that he did not experience (first-order inaccuracy) and takes himself to have experienced the represented event (meta-level inaccuracy).

Moreover, in both sorts of case, the meta-level *inaccuracy* is due to meta-level *error*. In “lost in the mall” cases, the inaccuracy arises because metacognitive monitoring misclassifies a first-order representation as having originated in experience. This might occur either because the subject applies inappropriate source monitoring criteria or because he applies appropriate source monitoring criteria that happen to be satisfied by the representation. In either case, there is meta-level error, but no meta-level *malfunction*: even a subject who reliably applies appropriate criteria may occasionally apply inappropriate criteria, and even appropriate criteria may occasionally misclassify a representation. In clinical confabulation cases, the inaccuracy arises because metacognitive monitoring fails to detect problematic first-order generation processes. This is meta-level error; it is not immediately clear whether it must result from meta-level malfunction. An approach in the spirit of Hirstein's (2005) would restrict the scope of confabulation to cases in which the subject applies inappropriate criteria and does so due to meta-level malfunction. Similarly, Gilboa et al. (2006) argue that confabulation occurs when subjects lose the “feeling of rightness,” impairing their ability to evaluate memory representations. Such an approach would rule out cases in which inappropriate criteria are applied (resulting in meta-level error) but in which this is not due to malfunction, as well as cases in which appropriate criteria are applied (but in which meta-level error nevertheless occurs). This approach may be overly restrictive, as it would classify as nonconfabulatory any condition in which malfunction is restricted to the first-order level. For example, if a subject with a malfunctioning memory system who would normally detect problematic first-order processes fails to do so on a particular occasion and therefore ends up with an inaccurate memory belief, classifying his condition as nonconfabulatory would obscure its similarity to paradigm cases of confabulation.

Thus it may be preferable not to require meta-level malfunction for confabulation. If so, then neither “lost in the mall” cases nor clinical confabulation cases are characterized by malfunction at the meta-level. We would thus have, in both types of case, first-order and meta-level inaccuracy, where the meta-level inaccuracy results from error which need not itself result from malfunction. Nevertheless, it is also easy to explain why the two types of case are distinct. In “lost in the mall” cases, when the subject initially imagines an event, he constructs a representation of an event that he did not experience, but he is not attempting to construct a representation of an event that he did experience. Here, inaccuracy does not result from error, and the question of malfunction does not arise. In clinical confabulation cases, in contrast, when the subject initially imagines an event, he constructs a representation of an event that he did not experience, but he is attempting to construct a representation of an event that he did experience. Here, inaccuracy results from error, and the error is due to malfunction. (In the case of SD, for example, the system recombines elements in an unreliable manner, i.e., in a manner unlikely to produce representations that are accurate with respect to the past.) In short, while the first-order representations at issue in both sorts of case are inaccurate, the inaccuracy in each case requires an entirely different explanation. The difference is between cases in which the failure results from a malfunctioning system and cases in which the failure is a mere byproduct of a properly functioning system.

#### 3.1.2. Simulationism and reliability

The type of memory error represented by “lost in the mall” cases—which results from metacognitive error but implies neither first-order nor meta-level malfunction—will be set aside until section 4. Focusing for now on confabulation proper, one might worry that, regardless of the plausibility of an understanding of confabulation in terms of malfunction, such an understanding is unavailable to the simulationist. As noted above, Robins—citing De Brigard's remark that “most of the time what you recall accurately depicts the witnessed event. Sometimes it does not. In both cases, however, the system is doing what it is supposed to do” (De Brigard, 2014a, p. 172)—claims that constructivists in general and simulationists in particular cannot appeal to malfunction in order to explain memory errors, since they “collapse the processing distinction between memory errors and successful remembering” (Robins, 2016a, p. 441). But this is true neither of De Brigard's particular version of simulationism nor of simulationism in general.

De Brigard's suggestion that “in both cases …the system is doing what it is supposed to do” appears to be due to rhetorical excess. It is true that, on his version of simulationism, inaccurate memory representations are an inevitable byproduct of the proper functioning of the system, due to its probabilistic character. But it is also true that he views the system as being designed to produce accurate representations most of the time. On this account, if the system (due, for example, to the sort of brain injury often responsible for clinical confabulation) begins to function in such a way that it no longer produces accurate representations most of the time, then it is *not* functioning properly, and De Brigard is not bound to say—and should not say—that it is doing what it is supposed to do.

Other versions of simulationism likewise do not collapse the processing distinction between memory errors and successful remembering. Causal theorists define the proper function of the system in terms of retention of information. Simulationists do not do so, but this does not mean that they cannot distinguish between proper function and improper function. Instead of defining the proper function of the system in terms of retention of information, they define it directly in terms of reliability. One version of simulationism that makes this explicit is Michaelian's. In Michaelian (2011b), he argued that, if the causal theory is to be able to distinguish accurately between successful and unsuccessful remembering, the causal condition must be supplemented by a reliability condition. As we will see below, however, there is reason to think that, once a reliability condition is added to the theory, the causal condition itself is no longer necessary. Thus, in Michaelian (2016b), he advocates replacing the causal reliabilist theory of memory with a pure simulation theory. A rough formulation of his version of the simulation theory says that to remember is to imagine (or simulate) the past. A more precise formulation, however, says that to remember is to imagine the past *in a reliable manner*. Thus, the pure simulation theory might just as aptly be called a pure reliabilist theory, and an understanding of confabulation in terms of malfunction fits well with the theory.

### 3.2. A provisional simulationist taxonomy

The definition of confabulation given above suggests a taxonomy of memory errors in which the retention condition, which requires retention of information from experience of the represented event, is replaced by a reliability condition requiring the reliable functioning of the episodic construction system (see Table 2). *Successful remembering* occurs when both the reliability condition and the accuracy condition are met. *Misremembering* occurs when the reliability condition is met but the accuracy condition is not. *Veridical confabulation* occurs when the reliability condition is not met but the accuracy condition is met. And *falsidical confabulation* occurs when neither the reliability condition nor the accuracy condition is met.

**Table 2:** **A provisional simulationist taxonomy of memory errors**.

	**Reliability**	**Accuracy**
Successful remembering	Yes	Yes
Misremembering	Yes	No
Veridical confabulation	No	Yes
Falsidical confabulation	No	No

The idea that misremembering satisfies the reliability condition has not yet been discussed. This is discussed below, but, before considering the place of misremembering in the taxonomy, it will be helpful to clarify the relationship between reliability and retention. A system functions reliably when it tends to produce mostly accurate representations, regardless of what grounds this tendency. If the causal theory of memory is right, the reliability of remembering is presumably due primarily to the fact that the system retains information from experience of remembered events. If the simulation theory is right, its reliability is at least in some cases wholly due to the way in which the system predicts or infers the features of past events.

There is nothing mysterious about the possibility of reliability without retention. Consider episodic future thought, the future-oriented counterpart to episodic memory. This is not the place to attempt to establish that imagining the future is reliable. But it might well be (see Michaelian, 2016a), and, if we suppose that it is, its reliability obviously cannot be explained in terms of retention, for the simple reason that, since future events have not (yet) been experienced, a subject cannot, when he imagines such an event, draw on information retained from his experience of it. The reliability of episodic future thought will, instead, have to be explained in terms of factors such as the heuristics on which the episodic construction system draws in order to predict the features of future events. Providing a full explanation along these lines would be no mean feat, but there is no obvious in-principle barrier to doing so. Similarly, there is no in-principle barrier to explaining the reliability of episodic memory in terms of factors other than the retention of information from remembered events.

Even if there is nothing mysterious about reliability without retention, it might seem that an explanation of reliability in terms of retention will be more straightforward than any explanation that the simulationist can offer. But this is not in fact the case. The analogy between memory and perception will again be helpful here.

#### 3.2.1. Retention without reliability

As noted in Section 2, just as the possibility of veridical confabulation can be invoked to motivate the causal theory of memory, the possibility of veridical hallucination can be invoked to motivate the causal theory of perception. It is, however, generally recognized that a causal condition by itself cannot provide an adequate theory of perception. Various sorts of causal connection might obtain between a perceptual representation and its putative object, and not all of these are capable of underwriting successful perception—some are “deviant.” The following case illustrates the point (Pendlebury, 1994). Suppose that Smith remains conscious while undergoing brain surgery. He continues to have visual representations, but these result exclusively from the surgeon's activities. A crow flies across the operating theater, startling the surgeon, who, as a result, bumps a microelectrode inserted in Smith's brain. By chance, this causes Smith to have a visual representation of a crow flying across the operating theater. Smith's representation is not only veridical with respect to the scene before his eyes but also caused by that scene. A simple causal theory of perception will therefore classify this as a case of successful perception. But despite the presence of a causal connection between his perceptual representation and the scene before his eyes, Smith is clearly not perceiving.

Parallel cases can easily be constructed for memory. Suppose that Jones witnesses an accident involving a red car in front of his office building but fails to encode any memory of the event. The accident leads the police to set up a roadblock, which, in turn, leads Jones to walk home from the office by a different route than usual. The route happens to lead past the office of a hypnotist, where Jones has himself hypnotized. The hypnotist implants in him a memory of having witnessed an accident involving a red car in front of his office earlier that day. When Jones arrives home and his wife asks him about his day, the implanted memory causes him to form a memory representation of an accident involving a red car occurring in front of his office building at the relevant time. Jones's representation is not only veridical with respect to the event but also caused by it. A simple causal theory will therefore classify this as a case of successful remembering. But despite the presence of a causal connection between his memory representation and the past event, Jones is clearly not remembering^7^.

The point is that, in order to rule out deviant causal chains, whether for perception or for memory, the causal condition must be supplemented with a further condition. Causal theorists of memory have often thought that memory traces can play this role. There is some controversy over the precise nature of memory traces (Sutton, 1998; De Brigard, 2014b). But regardless of how we understand the nature of memory traces, the thought is that a causal connection between an experienced event and a later memory representation of the event is sufficient for successful remembering only if it goes continuously via a memory trace: the trace must be produced by the experience, it must exist continuously during the interval between the time of the experience and the time of the later representation, and it must contribute to the production of the later representation. It is this thought that is expressed by Robins' retention condition, which in effect combines the causal condition with the memory trace condition.

The memory trace condition rules out the deviant causal chain in the hypnotist case just described. But causal theorists have not generally appreciated that simply requiring that the causal connection go continuously via a memory trace does not suffice to rule out all deviant causal chains. As long as we think of the trace as providing the full content of the later representation, it might appear to do so. But once we acknowledge—as do most contemporary causal theorists, including Robins—that the trace need not provide the full content of the later representation, it becomes clear that it does not. Take an ordinary case of confabulation. When asked what he did the day before, Smith, a subject with a malfunctioning memory system, generates an inaccurate representation of the day. Now modify the case so that a trace originating in his experience of the day makes a minor contribution to the production of his representation. Perhaps Smith spoke to his wife about a certain topic in a certain location. Due to the influence of the trace, his representation refers to a conversation with his wife. Due to the fact that his memory system is malfunctioning, however, it misrepresents the topic of the conversation and the location in which it took place. Despite the influence of the trace, this is clearly not a case of successful remembering. And it does not become a case of successful remembering if we modify it further so that the additional content generated by the confabulatory processes is (coincidentally) veridical, rather than falsidical. If this is unclear, we can reduce the contribution of the trace even further. Perhaps the trace is responsible only for Smith's representing that he did something with his wife. Perhaps it is responsible only for his representing that his wife wore a certain dress. At some stage, it will become clear that, despite the influence of the trace, Smith is not remembering. The point, again, is that the influence of a trace does not suffice to rule out deviant causal chains. Once this point is acknowledged—and it should be acknowledged by any causal theorist who acknowledges the reconstructive character of remembering, i.e., by any hybrid theorist—it becomes clear that we must add a further condition to the causal theory. The simplest such condition is a condition, along the lines of that discussed above, requiring the reliable functioning of the memory system.

#### 3.2.2. Reliability without retention

A number of causal theorists have come to a similar conclusion about perception, suggesting that the causal condition be supplemented with a reliability condition. Some have argued, moreover, that, once the reliability condition is adopted, the causal condition itself becomes redundant (see Kim, 1977). The idea here is that, since the reliability condition is satisfied in any case of successful perception, the causal condition does no work. Some have argued, in fact, that, once the reliability condition is adopted, it becomes clear that the the causal condition is not after all a necessary condition on perception.

The idea here is that there are hypothetical cases in which a subject perceives something even though his representation is not caused by it. Consider the case of a subject who seems to see through walls (Dretske, 1969). Smith seems to see through a tall, thick, opaque wall, on the other side of which various events unfold. When he faces the wall, the visual representations he has are indistinguishable from those he would have were the wall perfectly transparent. His visual representations are reliably correlated with the objects on the other side of the wall, but they are not caused by them. Intuitively, despite the absence in this scenario of any causal connection between Smith's representations and their candidate objects, Smith sees the objects. Or consider the case of a subject who seems to see the future (Johnston, 2004). Jones seems to see what will happen five minutes into the future in the direction in which he is currently facing. When he faces in a given direction, the visual representations he has are indistinguishable from those a normal subject would have when similarly positioned five minutes later. His visual representations are reliably correlated with events that will unfold five minutes into the future, but they are not caused by them. Again, intuitively, despite the absence of any causal connection between Jones's representations and their candidate objects, Jones sees the objects.

If we are moved by these cases, we might well conclude that, rather than supplementing the causal condition with a reliability condition, we ought simply to replace the former condition with the latter. Not all causal theorists will be so moved, however. And for good reason: the cases in question are purely hypothetical, and it is unlikely that we will be able to identify a single plausible but realistic case of perception without causation. In other words, there would appear to be no real-life counterexamples to the necessity of causation for perception. The situation with respect to memory, however, is rather different, for it is much more plausible to hold that there are real-life counterexamples to the necessity of causation for memory. Perception without causation is utterly mysterious. It is entirely unclear what sort of mechanism might, for example, enable a subject to literally see the future. Memory without causation is much less mysterious. Indeed, as we saw above, it need not be at all mysterious. While it is entirely unclear what sort of mechanism might enable a subject to literally see the future, it is much easier to see what sort of mechanism might enable him to reliably imagine the future. Much about the workings of this mechanism remains to be explained, but we have an increasingly good understanding of its basic principles (Michaelian et al., 2016). And a mechanism that enables us to reliably imagine the future might also enable us to reliably imagine the past. Thus, while a theory of perception that rejects the causal condition is not particularly plausible, a theory of memory that rejects the causal condition is far more plausible, and rejecting that condition is precisely what simulation theorists propose to do.

### 3.3. Misremembering

The simulationist defines misremembering in terms of reliability, rather than retention. According to the provisional taxonomy outlined above, misremembering occurs when the reliability condition is met but the accuracy condition is not. This distinguishes it from (veridical and falsidical) confabulation, in which the reliability condition is not met. Confabulation, we have seen, indicates malfunction. Misremembering, in contrast, is a byproduct of a properly functioning memory system. Few of us, fortunately, are prone to outright confabulation. But the sort of misremembering captured by the DRM paradigm is a routine occurrence and indicates no malfunction—it occurs when a reliable system happens to produce an inaccurate representation. In DRM studies, subjects who falsely recognize nonpresented lure words typically also successfully recognize many presented words.

Defining misremembering in terms of reliability, rather than retention, provides simulationism with a key advantage over causalism. The causalist sees misremembering as being characterized by retention and inaccuracy and falsidical confabulation as being characterized by lack of retention and inaccuracy. As we have seen, however, confabulation may sometimes involve a degree of retention. In practice, the causalist will thus have difficulty distinguishing between misremembering and falsidical confabulation. Because he sees misremembering as being characterized by reliability and inaccuracy and falsidical confabulation as being characterized by unreliability and inaccuracy, the simulationist, in contrast, is able to distinguish between misremembering and falsidical confabulation even where falsidical confabulation involves a degree of retention.

It is important to note that, while the simulationist rejects the causalist's definition of misremembering in terms of retention, simulationism is compatible with the view that remembering often does involve retention: the simulationist's claim is that remembering does not necessarily involve retention, not that it necessarily does not involve retention. This means that, while simulationists are bound to reject Robins' *definition* of misremembering in terms of retention, they are free to accept her *explanation* of the occurrence of misremembering in terms of retention. As Robins observes, the particular kind of inaccuracy that is involved in misremembering would appear to be explicable only on the assumption that the subject has retained information from the target event: an explanation of the fact that the subject in a DRM experiment falsely remembers a non-presented but thematically-consistent lure word seems to require that the subject has retained information about the theme of the presented words. Robins' challenge to the simulationist was to explain the occurrence of misremembering without invoking the retention of information. But this is not a challenge that the simulationist need take up. It is consistent with simulationism that, in every case of misremembering, the subject has in fact retained information from the target event. The simulationist argues that there are cases of successful remembering that do not involve retention of information, but he is free to say that some cases of successful remembering do involve retention; and he is free to say that the particular error of misremembering always involves retention. The simulationist is thus able to acknowledge the specific combination of success and failure that is characteristic of misremembering.

An additional advantage of the simulationist approach is that it better captures what misremembering has in common with other errors that do not involve a breakdown in reliability. On both De Brigard's and Michaelian's versions of simulationism, remembering is a probabilistic process, in the sense that the system attempts to predict the past on the basis of presently available information. This allows the approach to account for the kind of error involved in the DRM effect in a straightforward manner, since the presence of the falsely remembered word is likely given the presence of the other words on the list. But it also allows it to begin to account for the kind of error involved in “lost in the mall” cases, since the occurrence of the falsely remembered event is likely given that the subject now has a detailed representation of it.

## 4. The simulationist taxonomy

The provisional taxonomy outlined in Section 3 provides a plausible account of the relationships among successful remembering, misremembering, falsidical confabulation, and veridical confabulation. But it does not provide an account of the relationships between these forms of successful or unsuccessful remembering, on the one hand, and veridical and falsidical relearning, on the other hand. In order to provide such an account, an additional condition must be added to the taxonomy.

### 4.1. Relearning—the internality condition

As we saw above, relearning does not seem to be about accuracy or inaccuracy. When a subject forms a memory on the basis of reading a diary, for example, his memory representation might or might not be accurate. Nor does it seem to be about reliability or unreliability. When a subject forms a memory on the basis of reading a diary, his memory process might or might not be accurate. This will depend on a variety of factors, including the nature of diary (e.g., whether it contains mostly accurate information) and the manner in which he makes use of it (e.g., whether he is sensitive to the (in)accuracy of the information it contains). Instead, relearning seems to be about failure to satisfy an *internality* condition. Intuitively, veridical relearning occurs in cases in which the subject seems to remember, and to remember accurately, but in which he himself contributes no content to the retrieved memory representation; falsidical relearning occurs in cases in which the subject seems to remember, though to remember inaccurately, and in which he himself contributes no content to the retrieved memory representation. After relearning has occurred, of course, the subject may satisfy the internality condition and therefore subsequently remember or misremember; failure to satisfy the internality condition is characteristic of what happens at the time of relearning.

One way of satisfying the internality condition is to satisfy the retention condition, and causalists will be inclined to equate internality with retention, the thought being that what the subject contributes, in the case of remembering, or fails to contribute, in the case of relearning, is information retained from his experience of the target episode. As we have seen, however, the retention condition leads to difficulties with the distinction between relearning and confabulation. Moreover, satisfying the retention condition is not the only way of satisfying the internality condition. For simulationists, what the subject contributes, in the case of remembering, or fails to contribute, in the case of relearning, is indeed information, but this information need not be retained from his experience of the target episode. In many cases, the subject will indeed contribute retained information, but in other cases he may contribute only information generated during the reconstructive retrieval process. Similarly, in memory errors other than relearning, the subject contributes (retained or generated) information.

### 4.2. A revised simulationist taxonomy

**Figure 1:**
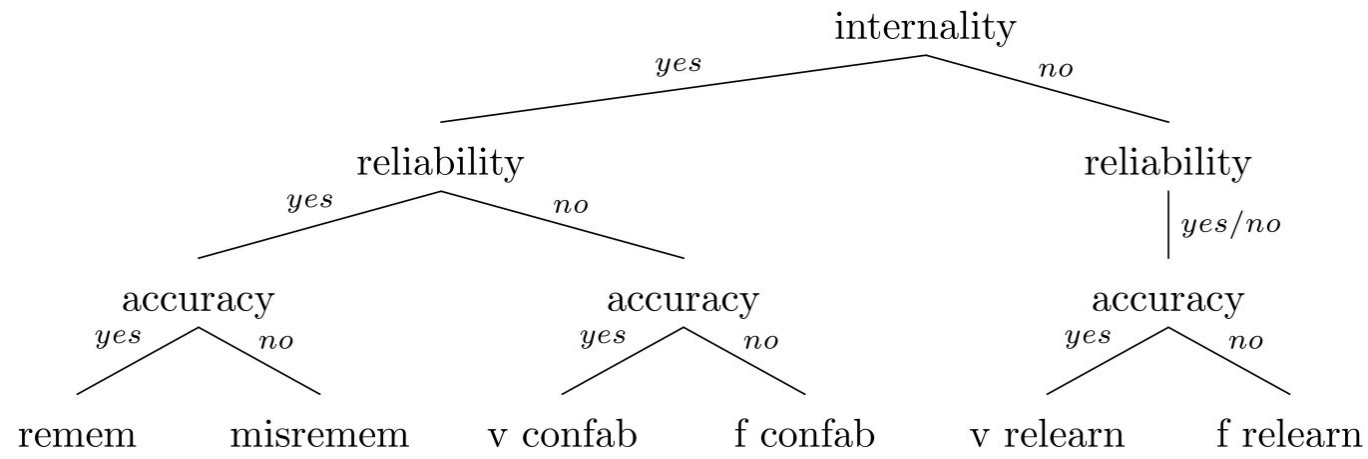
**The simulationist taxonomy of memory errors**.

Overall, then, the proposal is for a taxonomy which distinguishes among remembering and misremembering, veridical and falsidical confabulation, and veridical and falsidical relearning in terms of three conditions: accuracy, reliability, and internality. Successful remembering occurs when all three conditions are met. The various memory errors acknowledged by the taxonomy occur when one or more of the conditions is not met, in the pattern indicated in Table 3. Figure 1 provides a more vivid depiction of the relationships among remembering and misremembering, veridical and falsidical confabulation, and veridical and falsidical relearning.

**Table 3:** **The simulationist taxonomy of memory errors**.

	**Internality**	**Reliability**	**Accuracy**
Successful remembering	Yes	Yes	Yes
Misremembering	Yes	Yes	No
Veridical confabulation	Yes	No	Yes
Falsidical confabulation	Yes	No	No
Veridical relearning	No	Yes	Yes
Falsidical relearning	No	Yes	No
Veridical relearning	No	No	Yes
Falsidical relearning	No	No	No

This approach to memory errors was first hinted at in Michaelian (2016b), which suggested that confabulation might be distinguished from the sort of everyday memory errors that are exemplified by the DRM effect—what we have here been referring to as misremembering—in terms of reliability. Robins has recently objected to this suggestion, claiming, first, that the simulationist approach predicts that memory errors are more frequent in clinical subjects than in healthy subjects and, second, that it is unclear whether errors are in fact more common in clinical subjects than in healthy subjects.

Errors may be more common for clinical patients, or it may be only that the errors produced are more noticeable or that reports from such patients are met with more skepticism than everyday attempts at remembering. Determining how many attempted rememberings are errors, in either everyday or clinical cases, is difficult outside of controlled experimental conditions (Robins, Submitted).

This objection, however, rests on a misinterpretation of the role of reliability in the simulationist approach.

Robins interprets the approach as claiming that “it is when memory errors become more frequent—when they become the rule rather than the exception—that the system changes from functioning to malfunctioning. Michaelian's account thus allows us to say that the memory errors that occur in everyday cases [such as DRM errors] are consistent with memory's function because they are outnumbered by cases where remembering is reliable. Clinical confabulations, on the other hand, are malfunctions because these errors are a more common result of attempts at remembering” (Robins, Submitted). But this is not what the approach claims. Simulationism says nothing about the *frequency* of inaccurate representations in healthy or clinical *subjects* but rather something about the *tendency* of certain memory *processes* to produce inaccurate representations. What simulationism claims is that the process at work when a subject confabulates is unreliable, in the sense that it has a tendency to produce inaccurate representations. If a given subject uses that process most of the time, then he will under any realistic circumstances end up with mostly inaccurate representations. But a subject might do so more or less often, and so might end up with more or fewer inaccurate memory representations. Simulationism claims, further, that the process at work when a subject misremembers is reliable, in the sense that it has a tendency to produce accurate representations. If a given subject uses that process most of the time, then he will under any realistic circumstances have mostly accurate representations. But a subject might do so more or less often, and so might end up with more or fewer accurate memory representations. Thus, simulationism does not claim that clinical subjects necessarily have inaccurate memory representations more frequently than do healthy subjects, only that, when a clinical subject confabulates, the resulting memory representation tends to be inaccurate. In short, simulationism by itself makes no predictions about the frequency of inaccurate memory representations in clinical or healthy subjects. That being said, simulationism does predict that, if clinical subjects routinely confabulate, they will form inaccurate memory representations more often than do healthy subjects. While it may be difficult, outside of controlled experimental conditions, to determine whether this prediction is satisfied, that by itself does not tell against the simulationist taxonomy.

The simulationist taxonomy thus appears to be an improvement over the causalist taxonomy, but there is certainly room for further improvement. The role of metacognitive error, in particular, requires further investigation. As noted above, Hirstein (2005) views metacognitive error as essential to confabulation. That view can be challenged, but, regardless of whether metacognitive error ultimately turns out to be strictly speaking essential to confabulation, taking metacognitive error into account will complicate the taxonomy. If we take it into account, successful remembering will presumably require not only internality, reliability, and accuracy but also adequate metacognition. The type of error at issue in “lost in the mall” cases will occur when the internality, reliability, and accuracy conditions are met but the adequate metacognition condition is not. If metacognitive error is essential to confabulation, we will also have to acknowledge a type of memory error which is like confabulation but which does not involve metacognitive error. If metacognitive error is not essential to confabulation, we will have to consider the relationship between cases of confabulation that do satisfy the adequate metacognition and cases that do not. The role or lack thereof of metacognitive error in the other memory errors discussed here—misremembering and relearning—will also need to be investigated.

The errors discussed in the foregoing are all errors of *commission*, and the role of errors of *omission* likewise requires further investigation. Forgetting need not always amount to an error (Michaelian, 2011a). But when it does, the internality, reliability, and accuracy conditions would seem to be irrelevant. Instead, “erroneous forgetting” would seem to occur when the subject fails to produce a representation that he should have produced. Erroneous forgetting might thus be characterized not in terms of reliability, understood as a tendency to produce *mostly* accurate representations, but rather in terms of the related notion of power, understood as a tendency to produce *many* accurate representations (Goldman, 1986). The roles of both errors omission and metacognitive error are best left as questions for future research.

## Author contributions

The author confirms being the sole contributor of this work and approved it for publication.

### Conflict of interest statement

The author declares that the research was conducted in the absence of any commercial or financial relationships that could be construed as a potential conflict of interest.

## Footnotes

^1^Robins is concerned with confabulation only insofar as it is a *memory* error. The present article follows her in this, i.e., it is not concerned with nonmemorial forms of confabulation or with the relationship between nonmemorial and memorial forms of confabulation.

^2^What Robins refers to as misremembering might equally be referred to as false memory. Robins' term will be retained here, both because it nicely suggests that the error in question is possible only due to the subject's capacity to successfully recognize many presented items and because “false memory” can also be used to refer to errors (e.g., “lost in the mall” cases) from which DRM and similar errors are distinct. It is important to note that misremembering, as the term is used here, refers to memory for something that was not experienced, rather than failure to remember something that was experienced.

^3^While simulationism as such does not require the existence of a causal connection, underwritten by the retention of information, between the retrieved representation and the earlier experience, specific versions of simulationism may be able to incorporate modified versions of the causal condition. De Brigard's version of simulationism (discussed in greater detail below), for example, may be compatible with the claim that remembering necessarily involves a causal connection underwritten by modifications of synaptic connections, rather than retention of information in a more straightforward sense.

^4^In any real case of veridical confabulation, of course, the correspondence between the confabulatory representation and the relevant past experience is likely to be highly imperfect, but the same goes for many cases of successful (nonconfabulatory) remembering. And, no matter how stringent a standard of accuracy we adopt, the possibility of veridical confabulation will never vanish entirely.

^5^On many approaches, confabulation, strictly speaking, arises only in cases of amnesia due to brain lesions. Other approaches are available, but this article need not adopt a definite account of the mechanisms (or breakdowns of mechanisms) underlying confabulation: what matters, as far as the taxonomy is concerned, is that all standard approaches will agree that confabulation, whatever its underlying mechanism, always involves malfunction resulting in unreliability.

^6^When French et al. (2009) describe such cases as “a kind of confabulation in nonclinical subjects,” they explicitly distance themselves from Hirstein's definition.

^7^This is inspired by a case discussed by Martin and Deutscher (1966) but differs from it in making clear that there is a causal connection between the remembered event and the action of the hypnotist.
